# Biological Toxins as the Potential Tools for Bioterrorism

**DOI:** 10.3390/ijms20051181

**Published:** 2019-03-08

**Authors:** Edyta Janik, Michal Ceremuga, Joanna Saluk-Bijak, Michal Bijak

**Affiliations:** 1 Department of General Biochemistry, Faculty of Biology and Environmental Protection, University of Lodz, Pomorska 141/143, 90-236 Lodz, Poland; edyta.janik@unilodz.eu (E.J.); joanna.saluk@biol.uni.lodz.pl (J.S.-B.); 2 CBRN Reconnaissance and Decontamination Department, Military Institute of Chemistry and Radiometry, Antoniego Chrusciela “Montera” 105, 00-910 Warsaw, Poland; m.ceremuga@wichir.waw.pl

**Keywords:** biological toxins, bio-agents, biological warfare agents, bioterrorism

## Abstract

Biological toxins are a heterogeneous group produced by living organisms. One dictionary defines them as “Chemicals produced by living organisms that have toxic properties for another organism”. Toxins are very attractive to terrorists for use in acts of bioterrorism. The first reason is that many biological toxins can be obtained very easily. Simple bacterial culturing systems and extraction equipment dedicated to plant toxins are cheap and easily available, and can even be constructed at home. Many toxins affect the nervous systems of mammals by interfering with the transmission of nerve impulses, which gives them their high potential in bioterrorist attacks. Others are responsible for blockage of main cellular metabolism, causing cellular death. Moreover, most toxins act very quickly and are lethal in low doses (LD_50_ < 25 mg/kg), which are very often lower than chemical warfare agents. For these reasons we decided to prepare this review paper which main aim is to present the high potential of biological toxins as factors of bioterrorism describing the general characteristics, mechanisms of action and treatment of most potent biological toxins. In this paper we focused on six most danger toxins: botulinum toxin, staphylococcal enterotoxins, *Clostridium perfringens* toxins, ricin, abrin and T-2 toxin. We hope that this paper will help in understanding the problem of availability and potential of biological toxins.

## 1. Introduction

Bioterrorism is a form of terrorism that uses biological agents such as bacteria, viruses and toxins as weapons against humans, animals and crops. Like other forms of terrorism, the aim of bioterrorism is to intimidate the civilian population and authorities, as well as to accomplish the terrorists’ expected political, religious and ideological demands. The main effects of bioterrorism are diseases that often lead to death, contamination of water, food and soil [1]. What is more, such attacks cause mass panic and fear among the public, chaos in many spheres of life, economic losses and loss of faith in state authorities. The potentially massive number of victims exposes inefficiencies in the emergency services and healthcare system [2,3,4]. Biological agents used as biological weapons in bioterrorist activities are classified by the Center for Disease Control in the United States into three categories. Category A contain factors characterized by high pathogenicity and mortality, as well as ease of dissemination. These are: *Bacillus anthracis*, *Francisella tularensis*, *Yersinia pestis*, botulinum neurotoxins, haemorrhagic fever viruses (Ebola virus) and Variola major. Category B pathogens are moderately easy to spread, with low morbidity and mortality rates, but require specific monitoring and improvement of diagnostic capabilities. This category includes: ricin, abrin, *Brucella* species, *Clostridium perfringens* E toxin, and *Burkholderia mallei*. Category C includes emerging pathogens against which the population has little or no immunity and which could be engineered as bioweapons. These include the Hanta and Nipah viruses [5].

Toxins are biomolecules produced by bacteria, fungi, insects, plants, vertebrate and invertebrate animals, mainly for defensive purposes. These molecules induce detrimental effects in other organisms by inhalation, injection, ingestion or absorption [6]. Many of them affect the nervous system, disrupting the conduction of nerve impulses, for example by blocking the release of the acetylcholine neurotransmitter and ultimately the muscle contraction (botulinum toxin). At sublethal doses, the effect that this causes is temporary. Others toxins cause damage to cell membranes, and as a result lead to disturbances in the functioning of tissues and organs. The effects of such toxins are often irreversible and cause permanent damage to health. Compared to pathogens, biological toxins are also classified as bioterrorism factors, they are not infectious and do not replicate [7,8,9]. Toxins are significant in the health, food and in the security sector. Some of them are linked with natural intoxications. They are the etiologic agents of many food poisoning induced e.g., by staphylococcal enterotoxins [6]. However, they are extremely dangerous due to their lethal doses. A biological toxin’s lethal potential is measured in terms of the amount of material required to kill 50% of a group of test animals (usually rats or mice). This is written as LD_50_. LD_50_ < 25 mg/kg means that a substance is very toxic; LD_50_ < 25 mg/kg to 200 mg/kg is toxic; LD_50_ < 200 mg/kg to 2000 mg/kg < LD_50_ is harmful, while substances of LD_50_ > 2000 mg/kg are not classified as toxic agents [10].

Biological toxins have been used by humans for a long time. The first information on natural poisons and some guidelines on their preparation can be found in the Ebers Papyrus, which dates back to about 1500 B.C. This paper looks at many of the plants containing poisonous substances [11]. In 50 B.C. Dioskorides, in his Materia Medica, classified toxins to plant, animal and mineral toxins, depending on their origin [12].

Studies on the potential use of biological toxins in acts of bioterrorism have been carried out for years. One of the first units conducting research on the development of botulinum toxin as a biological weapon was the Japanese Unit 731, in Manchuria in the 1930s. During World War II, the United States also developed methods of mass-producing botulinum neurotoxin [13]. In the 1970s, T-2 mycotoxin was used in armed conflicts in Laos. The use of T-2 has resulted in death of more than 6000 people. There, it was dispersed in the form of a cloud of yellow dust. The toxic agent was presented as a yellow, oily liquid with a relatively large droplet size, which sounded like rain. As such, it was described as by witnesses as “yellow rain” [14]. In 1978, the Bulgarian dissident Georgi Markov was killed with ricin placed in the tip of an umbrella, which was then poked into the back of his leg [15]. Between 1990 and 1995, the Japanese Aum Shinrikyo sect, which had several thousand members repeatedly carried out a series of unsuccessful attacks using botulinum toxin [5]. The Iraqi Biological Program discovered by UNSCOM (UN Special Commission on Iraq) in 1995 (largely owing to the information disclosed by Hussain Kamel, an Iraqi general and a Saddam Hussain’s son-in-low, who escaped from Iraq) and described in detail by the Iraqis in the next of many Full, Final and Complete Disclosures in 1996 encompassed also production of many tons of botulin toxin and aflatoxin [16]. In 2013, an envelope of ricin was sent to the then-President of the United States Barack Obama [17]. In June of 2018, a plot involving ricin was foiled in Germany by authorities, the suspect had bought 1000 grains of ricin, a naturally occurring poison found in castor beans, and an electric coffee grinder online [18].

Biological toxins are very attractive to terrorists for use in acts of bioterrorism. The first reason is that many biological toxins can be obtained very easily. Simple bacterial culturing systems and extraction equipment dedicated to plant toxins are cheap and easily available, and can even be constructed at home. Many toxins affect the nervous systems of mammals by interfering with the transmission of nerve impulses, which gives them their high potential in bioterrorist attacks. Others are responsible for blocking the main cellular metabolism, causing cellular death [19,20]. The psychological effects of bioterrorism, must also be taken into account. Long-term anxiety may result from bioterrorist attack. Consequences of such anxiety could include mass sociogenic illness, mass panic and widespread behaviors that input pressure on emergency medical system, food and clean water supplies and public transport. In 2001 envelopes containing anthrax spores resulted in enormous fear, disruption of the mail service, distrust in the US government’s ability to protect its citizens. Hundreds of million dollars was spent on remediation with less than 22 clinical anthrax cases and 5 deaths [21].

Moreover, most toxins act very quickly and are lethal in low doses (LD50 < 25 mg/kg), which are very often lower than chemical warfare agents (Table 1).

For these reasons we decided to prepare this review paper which main aim is to present the high potential of biological toxins as factors of bioterrorism describing the general characteristics, mechanisms of action and treatment of most potent biological toxins. We hope that this paper will aid in understanding the problem of availability and potential of biological toxins.

### 1.1. Botulinum neurotoxins

**Figure 1:**
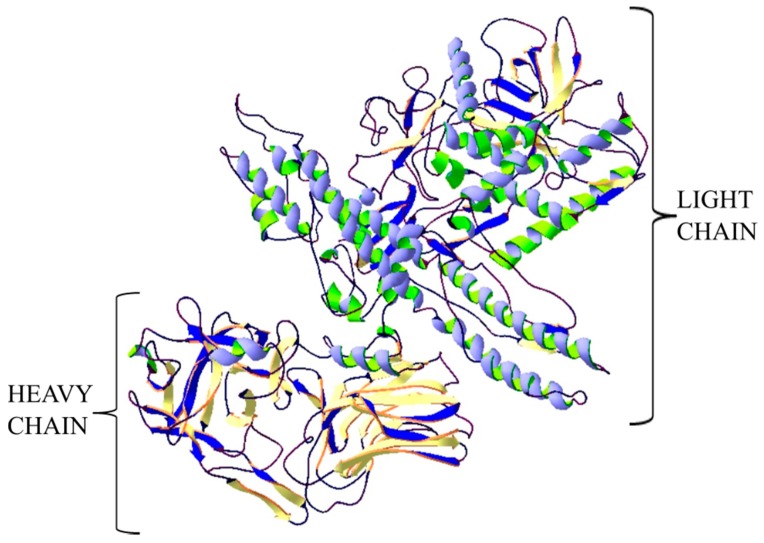
Structure of botulinum neurotoxin type A. The crystal structure of protein (PDB: 3BTA) [28] was taken from the RCSB PDB databank (http://www.rcsb.org/) (access on 05.03.2018). Visualization of the three-dimensional structure of the protein was performed using the Swiss-PdbViewer (http://spdbv.vital-it.ch/) (access on 05.03.2018.) [29].

Botulinum neurotoxins (BoNTs) are produced by the spore-forming, anaerobic Gram-positive bacteria of the genus *Clostridium* which consists more than 150 species. The disease caused by this toxin is called botulism. BoNTs comprise 7 serotypes (A to G) and more than 40 subtypes. However, A, B, C, E and F serotypes are responsible for inducing human botulism. In 2013, a new toxin produced by bivalent *Clostridium botulinum* strain was isolated from a human infant botulism case. Toxin was designated as BoNT/H also known as BoNT/FA and BoNT/HA. Genetic investigation of the toxin gene disclosed that BoNT/H shares ≈ 84% identity with BoNT/A1 in its binding domain, ≈ 80% with BoNT/F5 in its catalytic domain and displays a translocation domain similar to BoNT/F1. Another study showed that BoNT/H can be neutralized by currently used antisera. However, its toxicological profile is unusual because it displays lower potency and slow progression of botulism symptoms compared to primary BoNTs. Different investigations show that there are several resemblances between BoNT/H (i.e., BoNT/FA or BoNT/HA) and BoNT/F or BoNT/A and this is the cause of no consensus about the designation of the BoNT/H. [22,23,24]. The improvement of bioinformatics, data-mining tools and DNA sequencing techniques extended knowledge and understanding on the diversity of Botulinum neurotoxins. BoNTs and BoNT-related genes have been reported in bacterium *Enterococcus feacium* or *Weisseria oryzae*. These species may have the potential to produce BoNT-like toxins that show similar features in the multidomain organization of BoNTs but different toxicological features, specificity or operation principles [24]. BoNT/X is the first serotype of BoNTs identified by bioinformatics approaches and genomic sequencing. Toxin was identified in a *Clostridium botulinum* strain. This toxin displayed the typical BoNTs features including the residues forming a ganglioside binding pocket and metalloprotease consensus sequence or interchain disulphide bond. A remarkable feature of BoNT/X is its ability to cleave VAMP4 and atypical SNARE Ykt6. A new serotype, BoNT/X, BoNT/En and *Weissella oryzae* BoNT-like toxin (also known as BoNT/Wo) have been computationally-identified in recent years. The bioinformatics analysis of bacterium *Weisella oryzae* has led to the identification of an open reading frame 1 that has a strong sequence similarity with *bont* genes, but that lacks the additional genes usually associated within the *bont* locus in *Clostridia*. The sequence identity between BoNT/Wo and other BoNTs is ~14-16%. What is more the two cysteines that form the essential inter-chain disulfide bond in BoNTs are not conserved in BoNT/Wo, suggesting a different mode of action. A novel BoNT-like gene was discovered in the genome of *Enterococcus faecium*. This BoNT known as BoNT/En contain distinctive for Botulinum neurotoxins domain architecture, disulfide bond residues, conservation of the zinc peptidase HExxH motif. BoNT/En shows 29–38.7% identity with the other BoNTs [25,26]. Botulinum toxin has a protein structure and is one of the metalloproteins (zinc-dependent endopeptidases). BoNTs are synthetized as single polypeptides cleaved by clostridial or host proteases to its active form. The C-terminal heavy chain composes the binding and translocation domains of 50 kDa each, and is linked by a single disulfide bridge to the catalytic light chain forming the N-terminal part (Figure 1) [27]. 

This protein has three domains responsible for its biological functions. The binding domain is responsible for attaching the toxin to the receptor on the surface of the cell membrane of the target cells—nerve cells. The translocation domain is responsible for the transport of toxin through the plasma membrane into the cell, while the enzymatic domain (with proteolytic activity) causes the splitting of peptide bonds in the SNARE transmembrane protein (SNAP-25, VAMP-1/2, syntaxin-1/2) that are integral to vesicular trafficking and neurotransmitters release. Depending on the type of toxin, the proteolytic effect is directed at different SNARE proteins. BoNT A and BoNT E hydrolysis SNAP-25 membrane protein. Type B, F and G hydrolysis VAMP-1/2, while BoNT C hydrolysis SNAP-25 and syntaxin-1/2 [30,31,32].

The mechanism of the toxic action of botulin toxin inhibits the release of neurotransmitters, including acetylcholine, within neuromuscular junctions, resulting in relaxation and paralysis of skeletal muscles. The first symptoms occur within a few to 36 h of poisoning with the toxin. Irrespective of the type, there are similar clinical manifestations of poisoning. Initially these are speech and swallowing difficulties, double and blurred vision, anxiety, lack of saliva and tears. There then follows a loss of control over the body, and atrophy of the throat reflex appears. Respiratory muscle paralysis then causes respiratory failure, and this is the main cause of death of infected patients [33,34,35].

Botulinum toxin is calling the most toxic substance in the known world. It is estimated, that the mice LD_50_ for parenteral administration is 1 ng/kg [36]. As the only biological toxin, it has been classified by the CDC in Atlanta as a Category A bio agent. The lethal dose for a human weighing about 70 kg is 0.7–0.9 µg of inhaled toxin, or 70 µg of poison ingested with food. This toxin is not resistant to chemical and physical agents. It is depredated at 85 °C in 5 min, and is destroyed by sunlight within 1–3 h. Additionally, it is immediately decontaminated by chloride or H_2_O_2_ [37].

Treatment of botulism is based on the quickest administration of botulinum antitoxin, preferably 24 h after the first symptoms, because antitoxin neutralizes only toxin molecules that are not yet associated with nerve endings. The use of antitoxin can cause the occurrence of adverse reactions, such as post-surgical disease, anaphylaxis and hypersensitivity. Additionally, in most cases mechanically-assisted respiration and supportive treatment (with a return to self-reliance in up to 2-3 months), is also needed [35,38,39]. Strategies for the development of vaccines against botulism utilize two approaches. The first is to use a native BoNT to generate chemically- inactivated toxoid and the second is using recombinant techniques to engineer BoNT derivates [40]. Japanese scientist produced tetravalent botulinum toxoid vaccine derived from M toxin. Torii et al. demonstrated that the vaccine has the ability to induce neutralizing antibody above the level necessary for protection for those at risk. Study has demonstrated that the vaccine can provide immunity against botulism, is safe for the use in humans [41].

During a bioterrorist attack, botulinum toxin may be deployed in aerosol form or by contaminating water and food. The efficacy of the neurotoxin is equally high regardless of route of entry, due to having almost identical disease symptoms. A bioterrorist attack with botulinum neurotoxin is difficult to identify. Essentially, only an increase in the number of people with symptoms of toxin poisoning could indicate its use in a bioterrorist attack [42,43]. 

### 1.2. Staphylococcal Enterotoxins

Enterotoxins are a kind of exotoxin that is secreted by some pathogens. In nature, this group is mostly responsible for food poisoning. Enterotoxins include staphylococcal enterotoxins and AB5 group enterotoxins (i.e., cholera toxins), Shiga toxins and heat-labile enterotoxins produced by *Escherichia coli* [44]. However, the most potent of all these types of toxins have enterotoxin produced by *Staphylococcus aureus* [45].

Staphylococcal enterotoxins (SEs) are classified as as superantigens able to activate T lymphocytes (~20–30%), causing overproduction of cytokines, which are responsible for inflammation [46]. *Staphylococcus aureus,* which is responsible for toxin production, is a very rapidly spreading bacterium. Usually, the bacteria are present in the throat, nasal cavity, and crotch/anus. There is no vaccine for staphylococcus, and the disease itself is resistant to antibiotics. *Staphylococcus aureus* is often found in meat dishes and dairy products. Wu and Su studies demonstrate, that pre-cooked tuna meat as the potential to be contaminated with enterotoxin-producing *S. aureus* during the production of canned tuna products [47,48]. Its enterotoxins are mainly produced by *Staphylococcus* microorganisms, which are also present in food and cause food poisoning in humans. However, it is rare that they cause death. The symptoms persist for one or two days and mainly include abdominal pain, nausea, diarrhea and vomiting. They contain 21 serotypes: A, B, C1, C2, C3, D, E, G, G2, H, I, J, K, L, M, N, O, P, Q, R, S, T, U, U2 and V. Serotypes A, B, C1, C2, C3, D and E are known as ‘classic enterotoxins’, while the others are ‘new types’ [49,50].

**Figure 2:**
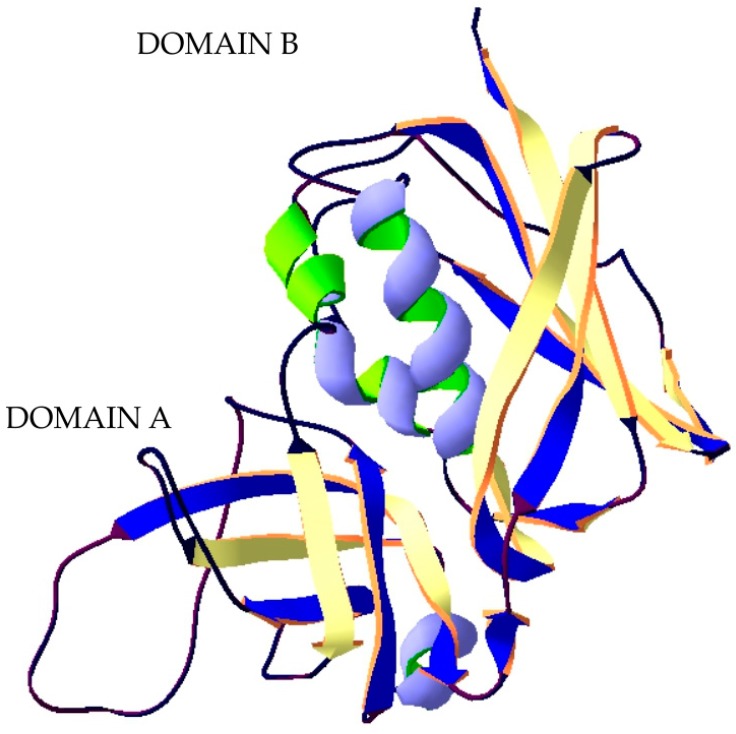
Structure of Staphylococcal enterotoxin B. The crystal structure of protein (PDB: 3SEB) [53] was taken from the RCSB PDB databank (http://www.rcsb.org/) (access on 08.03.2018). Visualization of the three-dimensional structure of the protein was performed using the Swiss-PdbViewer (http://spdbv.vital-it.ch/) (access on 05.03.2018) [29].

Staphylococcal enterotoxins are proteins with a mass of 26-35 kDa, having single polypeptide chains. SEB has 2 distinct unequal domains (composed mainly of β-strands and several α-helices) which are separated by a shallow groove and have a very complex tertiary structure (Figure 2). The smaller domain A These proteins are highly soluble in both water and salt solutions. Staphylococcal enterotoxins are highly resistant to proteolytic enzymes (pepsin, trypsin, rennin, papain), exhibiting unchanged biological activity in the gastrointestinal tract. These toxins are also resistant to gamma radiation, a wide pH range (2 < pH < 12), and dehydration. High thermal stability is characteristic of staphylococcal enterotoxins, which makes them potential food poisoning agents [51,52]. 

All genes coding for SEs are located on accessory genetic elements such as plasmids, prophages, genomic island vSa, *S. aureus* pathogenicity islands (SaPIs). Many of these are mobile genetic elements that is why their expansion among *Staphylococcus aureus* isolates can contribute to the evolution this pathogen or can change the ability to cause of diseases [52]. Superantigens are presented by class II MHC (Major Histocompatibility Complex) molecules but MHC peptide-antigen binding groove is not involved. They bind to conserved amino acid residues of MHC molecules outside of their peptide binding grooves and interact mostly with the Vβ domains TCRs (T-cell receptors). The result is stimulation of the ~20–30% entire population of lymphocytes T. Consequently, superantigens initiate systemic release of chemokines and proinflammantory cytokines that led to diverse immune-mediated diseases for example toxic shock syndrome. T lymphocytes are subjected to apoptosis or enter a state of energy. Natural food poisoning is usually caused by A and D staphylococcal enterotoxins, but less frequently also by the B and C types [54,55].

The most dangerous enterotoxin, with great potential for use in bioterrorism, is Staphylococcal Enterotoxin B (SEB). What is more that is the only *Staphylococcus* enterotoxin that has been examined as a biological weapon. It is the most heat resistant toxin, and is detected even in samples of thermally sterilized food. Enterotoxin B induces toxic shock syndrome in the body, which is a very strong immune response by the human organism. Strong food poisoning occurring very quickly can lead to dehydration and death. In the case of SEB being ingested into the body other than through food, for example by inhalation, it can trigger a septic response throughout the organism. The toxicity parameters for this type of poisoning are LD_50_ 20 µg/kg and ED_50_ 400 ng/kg. Treatment is mainly based on conservative treatment, while inhalation intoxication is treated through the administration of anti-inflammatory steroids [51,56,57].

### 1.3. Clostridium perfringens Toxins

*Clostridium perfringens* is a Gram-positive, anaerobic, spore-forming, rod-shaped, bacterium common in many different microbiotas, and are found in the soil, marine sediment, in decaying vegetation, and in the intestinal tract of humans and other animals [58,59]. *C. perfringens* is considered to be the most widespread pathogenic bacterium in the world [60]. These bacteria are able to produce more than 15 different toxins. However, four of them (alpha (CPA), beta (CPB), epsilon (ETX) and iota (ITX)), are the major toxins and have high toxic potential. The Alpha and epsilon toxins have been classified by the CDC as Category B bioterrorism agents, which suggests their potential in these kinds of acts [61,62,63]. 

CPA is a 43 kDa protein containing two domains, an alpha-helical N-terminal domain harboring the phospholipase C active site, and an alpha-sandwich C-terminal domain involved in membrane binding. This toxin is a classic example of a toxin that modifies cell membranes through enzymatic activity. CPA is zinc-dependent phospholipase C which hydrolyzes sphingomyelin (SM) and phosphatidylocholine (PC) in the eukaryotic cell membranes. Furthermore, SM metabolism triggered by low CPA doses is related with the production of pro-apoptotic mediators including N-acylethanolamine, saturated fatty acid and release of mitochondrial cytochrome C, caspase-3 activation and led to apoptosis of the cells. This damages the cell membrane which results in cell lysis. Additionally, the lipolysis of the cell membrane activates an arachidonic cascade resulting in the formation of thromboxanes, leukotrienes and prostaglandins responsible for activate the inflammation cascade and produce vasoconstriction. This toxin can reduce leukocytes migration and promotes their aggregation by different mechanisms such as hyperadhesion with leucostasis at the periphery of lesions. What is more, CPA in combination with theta toxin allows *Clostridium perfingens* to escape macrophage phagosomes and survive in the host organism. The ensuing intravascular haemolysis and capillary damage, platelet aggregation and hepatic necrosis results in multiple organ failure [64]. 

ETX is the third most potent biological toxin after botulinum toxin and tetanus toxin, with an LD_50_ of about 70 ng/kg. ETX contains three domains that are mainly composed of β-sheet. First domain consists of α helix followed by a loop, and three short α helices. A cluster of aromatic residues (Tyr42, Tyr43, Tyr49, Phe212) in this domain can be responsible for in receptor binding. Second domain contains two stranded sheets with an amphipathic sequence and this domain can be related with channel forming. Third domain is a β-sandwich and contains the cleavage site for toxin activation [60]. The active toxin is a protein with a molecular mass of 29 kDa, with relative resistance to proteases in the gastrointestinal tracts of mammals. This toxin is most stable at room temperature for up to a few weeks, and far longer at lower temperatures [65]. ETX interacts with the cellular membrane and creates pores within the membrane that modify its porousness and quickly becomes cytotoxic. Epsilon toxin induces pore formation in eukaryotic cell membranes via detergent-resistant, and cholesterol-rich membrane domains (lipid rafts). This damage results in very fast degenerative and necrotic changes in cells, leading to organ failure [66]. ETX can also cross the blood-brain barrier binds to myelin structures causing death of oligodendrocytes and inducing central nervous system demyelination. Epsilon toxin acts on a several cell lines including Madin–Darby canine kidney (MDCK), human renal leiomyoblastoma (G-402) and mouse kidney cells (mpkCCD_C14_) which have a specific receptor for ETX. In this cell line toxin binds to the cell surface and recognizes a putative specific membrane receptor which not occurs in insensitive cells. The cytotoxicity in MDCK cells is a result of pore formation and causes loss of intracellular K^+^, increase Na^+^ and Cl^−^ [60]. *C. perfringens* can cause a wide range of diseases including, amongst others, gas gangrene in man and necrohemorrhagic enteritis in suckling and veal calves. Most of these diseases follow a very rapid, often fatal course. Therefore, curative treatment is difficult and control must rely on preventive measures, including vaccination [60]. The greatest potential for this toxin’s use in bioterrorism is in an aerosolized form that can be used as a bioterrorist weapon. Additionally, this toxin can be dispersed in food intended for human consumption [67]. 

**Figure 3:**
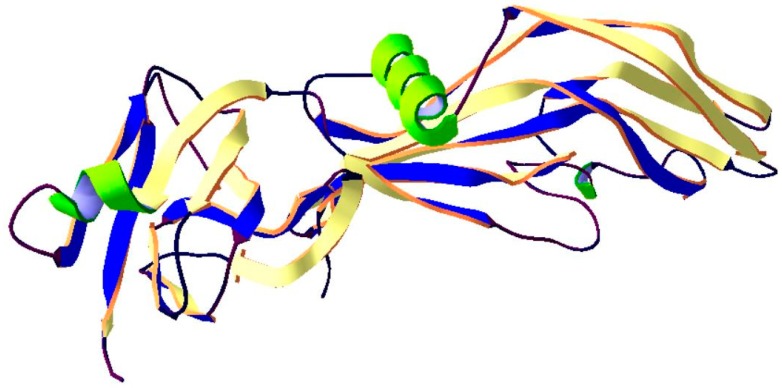
Structure of C. Perfringens enterotoxin. The crystal structure of protein (PDB: 3AM2) [68] was taken from the RCSB PDB databank (http://www.rcsb.org/) (access on 22.02.2019). Visualization of the three-dimensional structure of the protein was performed using the Swiss-PdbViewer (http://spdbv.vital-it.ch/) (access on 05.03.2018). [29].

Nevertheless, some *C. perfringens* strains, mostly type A are able to produce another medically important *C. Perfringens* enterotoxin (CPE) CPE producing *C. perfringens* is a major cause of *Clostridium perfringens* type A food poisoning and non-food-borne human gastrointestinal diseases like a sporadic diarrhea, nosocomial diarrheal diseases and antibiotic-associated diarrhea. CPE damages intestinal epithelia by pore-forming mechanism disrupting the selective permeability of the plasma membrane. This toxin consists of a single chain polypeptide of 319 amino acids which has an elongated shape, composed of three distinct domains, I, II, and III (Figure 3). The first domain is responsible for binding to the specific receptor claudin—most important components of the tight junctions. The other domains (II and III) are responsible for creating a pore-forming module. Domain II and domain III have three short β-strands each, by which they are distinguished. Additionally, in domain II is an α-helix lying on the β-strands. The sequence of amino acids composing the α-helix and preceding β-strand is characteristic for transmembrane domains which are forming β-barrel-made pores. There is a hypothesize that the transmembrane domain of CPE is inserted into the membrane upon the buckling of the two long β-strands spanning the module, a mechanism analogous to that of the cholesterol-dependent cytolysins [68].

### 1.4. Ricin

Ricin is a phytotoxin presented in seeds of *Riccinus communis*. It can be obtained both from the native seed as well as waste product obtained during extrusion of castor oil [69]. The castor plant grows wild in tropical and subtropical climates and the plant is cultivated in large scale for the commercial production of castor oil, a major component of the castor bean, with almost two million tons of seeds harvested annually. The major components of castor beans include lipids, proteins and carbohydrates, whereas ricin constitutes about 1% of the seed dry weight [70]. Ricin is a member of the AB superfamily of plant and bacteria protein toxins that exploit retrograde transport as a means to gain entry into the cytosol of host cells [71]. Pure, extracted ricin is an environmentally stable, white-yellow powder. Ricin can be prepared and remain stable in various forms: liquid, crystalline, and even aerosol [72]. Ricin was discovered by Hermann Stillmark, who extracted the toxin from castor seeds. During the study, the scientist also observed that ricin causes agglutination of erythrocytes and precipitation of serum proteins Ricin is stable in the environment, however, it can be inactivated by heating at 80 °C for 10 min, or at 50° C for 1 h. Ricin is also sensitive to chlorine solutions [99.8% inactivation by 100 mg/L free available chlorine (FAC) for 20 min]. The toxin will remain stable in low chlorine concentrations (10 mg/L FAC) and in iodine at up to 16 mg/L [17].

**Figure 4:**
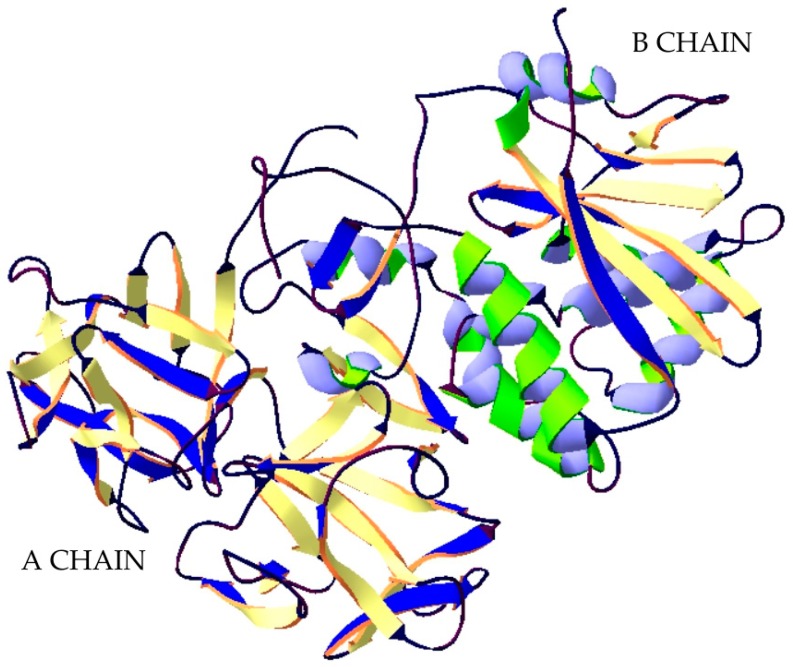
Structure of Ricin. The crystal structure of protein (PDB: 2AAI) [75] was taken from the RCSB PDB databank (http://www.rcsb.org/) (access on 06.03.2018). Visualization of the three-dimensional structure of the protein was performed using the Swiss-PdbViewer (http://spdbv.vital-it.ch/) (access on 05.03.2018). [29].

Ricin is part of a group of proteins called lectins. A lectin is a protein that has a strong affinity to sugar residues. Ricin is a 60–64 kDa heterodimer consisting of two protein chains: A and B, connected to each other by a disulphide bridge (Figure 4). The B chain is a galactose-specific lectin that binds to mannose containing glycoprotein, which is found in the outer layer of the cell membrane, and facilitates the endocytosis of the ricin to the endoplasmic reticulum (ER). Within the ER lumen, a resident protein disulfide isomerase facilities reductive separation of A and B subunit. Chain A exhibits N-glycosidase RNA activity and depurinates an adenine residue of the 28 S ribosomal RNA loop contained within the 60 S subunit. This irreversible process inactivates elongation of polypeptides and leads to cell death. It is believed that Chain A exploit cellular pathways that function to transport misfolded proteins through the endoplasmic reticulum membrane into the cytosol for degradation by proteasomes. Next utilizes specific components of the ER associated degradation pathway to dislocation [73,74].

The result is the inactivation of ribosomes in the cell and the blocking of protein synthesis, leading to cell death. A single ricin A chain is capable to inactivate approximately 1500 ribosomes per minute [69]. Toxicity results from the inhibition of protein synthesis, but other mechanisms are noted including apoptosis pathways, direct cell membrane damage, alteration of membrane structure and function, and release of cytokine inflammatory mediators [76].

The clinical manifestations of toxin poisoning depend on the route of entry into the organism. Following ingestion of castor bean plant seeds or food contaminated with the toxin, the alimentary tract is the first to be affected and damaged. The symptoms do not occur immediately upon ingestion but after a few days of vomiting and diarrhea, as a result of the alimentary tract irritation. This causes bodily dehydration, which is followed by alimentary tract bleeding and necrosis of the liver, kidneys and pancreas. Vascular collapse tends to follow next. Intramuscular administration of the toxin causes edema in the injection area and necrosis of local lymph nodes, while other typical symptoms include gastrointestinal bleeding and renal necrosis. If the toxin enters via the respiratory tract, the symptoms that occur within several hours following inhalation include fever, coughing and progressive respiratory insufficiency and tightness in the chest. In more severe cases, pulmonary edema, hypotension and vascular collapse are observed. [77,78]. Pulmonary ricin intoxication is considered most hazardous [79]. No effective treatment is available for toxin intoxication [80]. Treatment is mainly supportive and minimizes the effects of the poisoning. Treatment consists of hydration of the organism, restoration of the proper level of electrolytes, and respiratory support. Dialysis is not effective. Victims of ricin poisoning are not dangerous to their environment, because the illness it causes cannot be spread from person to person There is no American FDA-approved medication for ricin poisoning. However, there is a vaccine called RiVax, based on genetically inactivated subunit-A chains. Immunogen is enzymatically inactive and there is no residual holotoxin toxicity. Tests on animal models show high efficacy and safety of use in humans [33].

Ricin can be used in acts of bioterrorism in several forms, as it has many ways to penetrate the organism. It can be used as an aerosol and by poisoning water and food. The toxicity of ricin depends on different factors, such as the route of entry into the body, the dose and species (Table 2). The LD_50_ value for humans after ingestion is estimated at about 22–25 µg/kg body weight. Just 5-6 grains of castor bean plant seeds are considered a lethal dose to children, while for adults it is about 20 grains [81,82,83,84].

Ricin as also characterized by its stability in the environment and relative ease of extraction. Despite having a well-understood mechanism of action, treatment is still based on supporting the organism, which in a bioterrorist attack with a high casualty count, can cause chaos in the local healthcare system. As such, more research is needed on more accurate, effective treatments [82]. Due to its ease of production, good stability, availability and high lethality, ricin exhibits potential as a biological weapon. Ricin is listed in the US Centers for Disease Control (CDC) as a second highest priority biothreat agent [85].

### 1.5. Abrin

Abrin same as ricin is a phytotoxin isolated from the *Abrus precatorius.* Plant is commonly known as the ‘rosary pea’ or ‘jequirity bean’ [86]. This plant is indigenous to is found in South Africa, China, West Indies, India, Brazil, but is also now widespread and can be found in other parts of the world, such as Florida, Alabama, Georgia and Hawaii in the United States, and Puerto Rico [87]. Abrin can be use by aerosolization as a dry powder or liquid droplets, or by addition to food and water as a contaminant [88].

**Figure 5:**
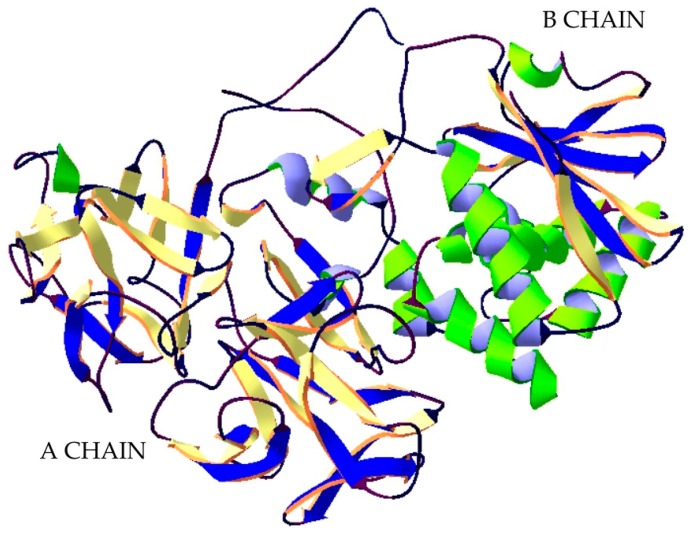
Structure of abrin. The crystal structure of protein (PDB: 1ABR) [91] was taken from the RCSB PDB databank (http://www.rcsb.org/) (access on 05.03.2018). Visualization of the three-dimensional structure of the protein was performed using the Swiss-PdbViewer (http://spdbv.vital-it.ch/) (access on 05.03.2018) [29].

Abrin is a protein with molecular mass of approximately 60–65 kDa. It is also heterodimer consisting of two disulfide-linked polypeptides, known as the A–chain and B-chain (Figure 5) [89]. The B chain (30 kDa, 263 amino acids), which has the properties of lectin, acts as a binding domain. Its task is to connect to the glycoprotein receptors on the cell surface. Toxin is transferred into the cell through endocytosis. A chain (28 kDa, 251 amino acids) is also RNA N-glycosidase (like ricin). Its mechanism of action also consists in blocking the process of translation into the penetrated cells. In the cells’ cytosol, chain A cleaves the C-N bond in adenines, located in the small subunit of the ribosome 28S rRNA at the position A4324. Adenine depurination destabilizes the rRNA preventing the binding of ribosomes to elongation factors, and this results in inhibition of protein synthesis [90].

Abrin has the similar structure, properties and mechanism of toxic action with ricin, but it is 75 times more toxic than ricin [92]. Abrin poisoning as a result of ingestion of seeds or contaminated food causes severe abdominal pain, vomiting and diarrhea. These symptoms result in kidney failure. In most cases, bleeding from the gastrointestinal tract is also observed. If abrin enters the organism as a result of inhalation, symptoms such as pulmonary edema, hypertension in the pulmonary arteries and hemolysis of red blood cells will appear. Death usually occurs 36-72 h after exposure, depending on the route of entry and dose. Because there are no drugs or vaccines available against abrin poisoning, the treatment is supportive and based on minimizing the effects of the poisoning. The type of medical care depends on several factors, the most important being based on the route of infection. Measures include respiratory support, intravenous fluid administration and blood pressure stabilization. Possible neurological symptoms after exposure include hallucinations, reduced consciousness and convulsions. Recognition of the poisoning as well as its medical treatment are identical to the response to ricin poisoning [93].

Abrin is the strongest plant toxin, which means it can be potentially used as a biological weapon. *Abrus precatorius* seeds are very popular around the world. They are used as rosary beads, and beads in bracelets and other jewelry. This makes access to abrin easy to obtain. Abrin toxin can be used in several forms: aerosol, pellets or dissolved in water. Abrin is about 30 times more toxic than ricin. There are no reported cases of the use of abrin in bioterrorist activities, but natural poisoning with abrin indicates its high potential for such use [9]. Owing of its stability, extreme toxicity and ease of purification abrin is classified as select agent by CDC. LD_50_ dose is only 0.04 µg/kg body weight of mice [94]. Estimated fatal dose for humans is 0.1–1 µg kg ^−1^ body weight. No antidote or vaccine for abrin has been described except a neutralizing antibody inhibits abrin toxicity both in vitro and in vivo [95].

### 1.6. T-2 Toxin

T-2 toxin is a one type-A trichothecene mycotoxin, produced by the metabolism of *Fusarium species*, such a *F. tricinctum, F. sporotrichiella*, *F. sporotrichioides, F. poae* and *F. sulphureum.* T-2, which is harmful to human health and animal husbandry, is often found in cereals, particularly in oats, barley, wheat, feed and animal feed, especially in the cold climate or wet storage conditions. Pleadin et al. reported that in unprocessed cereals, food and feed coming from Croatia and Bosnia & Herzegovina, the incidence of T-2 toxins, ranged from 26.9% to 81.6% [96]. In Kashin-beckdisease (KBD) endemic villages in China, T-2 toxin contamination is relatively high with an average level of 78.91 µg/kg in wheat and 47.47 µg/kg in flour [97].

**Figure 6:**
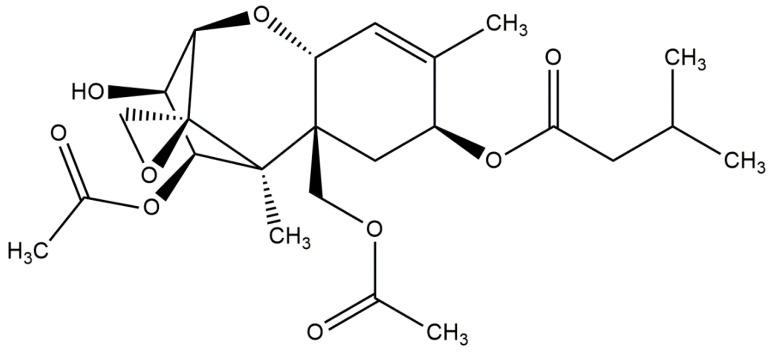
T-2 toxin chemical structure (structure generated from Isomeric SMILES code available on https://pubchem.ncbi.nlm.nih.gov/) (access on 15.05.2018).

Chemically, T-2 toxin is a low-molecular organic compound with a mass of 466 Da. The structure of this molecule is a tetracyclic sesquiterpenoid 12,13-epoxytrichothec-9-ene ring in common, and a 12,13-epoxy ring responsible for the toxicological activity. Its chemical structure is characterized by a hydroxyl (OH) group at the C-3 position, acetyloxy (-OCOCH3) groups at C-4 and C-15 positions, an atom of hydrogen at C-7 position and an ester-linked isovaleryl [OCOCH2CH(CH3)2] group at the C-8 position (Figure 6) [98]. This chemical structure is part of the Trichothecenes group. This group includes various toxins such as neosolaniol, saratoxin, diacetoxyscirpenol, crocetin and HT-2. However, T-2 possesses the highest potential [99].

T-2 toxin is resistant to heat and ultraviolet light-induced inactivation, and is very stable and relatively insoluble in water [97]. T-2 toxin has a wide toxic effect on the organism. The mechanism of action of this molecule stems from the presence of an epoxy ring in the structure, which is capable of intracellular reactions with nucleophilic groups. T-2 reacts with membrane phospholipids, damaging cellular structures [100]. This mycotoxin has the high affinity for the 60S ribosomal subunit, resulting in inhibition of the activity of peptidyltransferase. Therefore, the primary known toxicity mechanism of the T-2 toxin is its inhibitory effect on protein synthesis. Additionally, some research has reported that T-2 toxin can affect DNA and RNA synthesis, mitochondrial electron transport system, mitochondrial function, and cell division and membrane function [97]. T-2 mycotoxin is about 400-fold more potent than sulphur mustard in producing skin injury. Dermatotoxic effects of T-2 toxin are characterized as enterohaemorrhagic dermatitis. T-2 produces edema, intradermal hemorrhage and necrosis of the skin [101]. It’s also toxic to the digestive system, nervous system, genital dermis and is teratogenic and carcinogenic [102]. T-2 also targets the immune system. Several studies described that this mycotoxin could decrease the production of interleukin (IL)-2 and the expression of plasma interferon-gamma (IFN-γ) and can upregulate the mRNA expression of IL-6, IL-1β and tumor necrosis factor-α (TNF-α) in a dose-dependent manner in RAW264.7 cells [97].

T-2 mycotoxin can penetrate into the body with food, or as a smoke or aerosol sprayed by different dispersive systems. The first symptoms of T-2 mycotoxin poisoning can appear just a few minutes after exposure [98]. Severe poisoning results in prostration, weakness, ataxia, collapse, reduced cardiac output, shock and death. Meningeal hemorrhage in brain and nervous disorders has been reported for T-2 mycotoxin exposure in humans [101]. Exposure to this Trichotecene induces also inhibition of erythropoiesis in bone marrow and spleen [103]. Because of the lack of drugs for treating poisoning with T-2 mycotoxin, treatment is based on maintaining the functions of the respiratory, circulatory and digestive systems. Activated charcoal is recommended for removing the toxin from the gastrointestinal tract. In the case of skin contact with mycotoxin, it is necessary to remove it from the skin’s surface [104].

T-2 toxin has a high mortality rate. LD_50_ is approximately 1 mg/kg body weight. In addition, this mycotoxin has a strong irritant effect on the skin and also causes systemic toxicity, which is not characteristic of biological toxin [105]. Because of its anti-personnel properties, ease of large-scale production, and apparent proven delivery by aerial dispersal systems, the T-2 mycotoxin has an excellent potential for weaponization. An accidental exposure of T-2 extract (200 mg/kg) to two laboratories workers is also reported with similar symptoms, which lasted for 4–5 days. Based on above published reports the possibility of human exposure to this mycotoxin at higher concentrations by dermal exposure either by intentional use (bioterrorism), accidental or by handling contaminated agricultural products cannot be ruled out [106]. The many possible routes of toxin ingress into the organism make it a potential biological weapon. A potential bioterrorist could use food and water or other systems to disperse mycotoxin, e.g., in the form of aerosols or droplets, in order to poison the population. It is important to note that the T-2 toxin has already been used in armed conflicts, and that thousands of people have died as a result. The symptoms of toxin poisoning appear quickly and death occurs within a few minutes, hours or days. An additional attribute of T-2 mycotoxin as a biological weapon is the lack of medicine and detailed treatments, which can significantly delay or prevent the recovery of humans exposed to T-2 toxin [104].

T-2 toxin, considering the lack of accurate data on its action mechanisms in the human body, could become a perfect B weapon. Moreover, studies of T-2 have not gone beyond the laboratory level and information on its lethal doses or effects are estimates and based on animal reactions. Another advantage of the toxin in biological warfare is the lack of drugs counteracting it, which greatly delays or prevents recovery. Additionally, this toxin was used in biological warfare in Iraq (by Saddam Husain), as well as by Soviet forces in Kampuchea and Afghanistan (1979-81). There were also reports of “yellow rain” in Laos during the Vietnam War [107].

## 2. Conclusions

Many toxins produced by various plants and microorganisms are highly toxic. Therefore, they may be potential biological weapon for terrorist or military use. Some biotoxins, such as botulinum neurotoxin, are used as therapeutic agents and have a wide spectrum of effects. BoNT has been used in the treatment of cervical dystonia, eyelid contracture and migraine. However, the characteristic properties of these types of compounds, like a high toxicity, ease of production and stability in the environment, all favor their use in bioterrorist attacks. Toxins as a botulinum neurotoxin, ricin and abrin are a unique group of compounds that have been listed by the United States’ CDC as potential agents of biological weapons. Bioterrorism and its effects can impose heavy demands on the public health care system which will be called upon to handle the consequences. It is important to develop effective countermeasures to enable rapid epidemiological and laboratory investigation, disease surveillance and efficient medical management. Knowledge about the clinical symptoms associated with the poisoning of highly dangerous toxins is also important, in order to understand the mechanisms of action of individual biotoxins and thus improve our general knowledge of them. 

## Figures and Tables

**Table 1:** Comparison of biological toxins toxicity with chemical warfare agents.

Agent	LD_50_ Parameter (µg/kg)	Molecular Weight (Da)
Botulin toxin	0.001–0.002	150,000 (Protein)
Shiga toxin	0.002	55,000 (Protein)
Tetanus toxin	0.002–0.003	150,000 (Protein)
Abrin	0.01–0.04	65,000 (Protein)
Ricin	0.1–1	65,000 (Protein)
Clostridium perfingens toxins	0.1–5	35,000–40,000 (Proteins)
VX	15	267
Staphylococcal enterotoxin B	27	25,000 (Protein)
Soman	64	182
Sarin	100	140
Aconitine	100	647
T-2 mycotoxin	1210	466

**Table 2:** Lethality of ricin (based on [83]).

Exposure	Inhalation	Intravenous Injection	Ingestion	Subcutaneous Injection
Human LD_50_	3 µg/kg	3 µg/kg	22–25 µg/kg	500 µg/kg
Mice LD_50_	3–5 µg/kg	5 µg/kg	20 mg/kg	24 µg/kg
Time to death	36–72 hrs	36–72 hrs	6–8 days	3–4 days
